# Decision curve analysis: confidence intervals and hypothesis testing for net benefit

**DOI:** 10.1186/s41512-023-00148-y

**Published:** 2023-06-06

**Authors:** Andrew J. Vickers, Ben Van Claster, Laure Wynants, Ewout W. Steyerberg

**Affiliations:** 1 Department of Epidemiology and Biostatistics, Memorial Sloan Kettering Cancer Center, New York, NY USA; 2 Department of Development and Regeneration, KU Leuven, Louvain, Belgium; 3 Department of Biomedical Data Sciences, Leiden University Medical Center, Leiden, the Netherlands; 4 EPI-Centre, KU Leuven, Louvain, Belgium; 5 Department of Epidemiology, CAPHRI Care and Public Health Research Institute, Maastricht University, Maastricht, Netherlands

## Abstract

**Background:**

A number of recent papers have proposed methods to calculate confidence intervals and *p* values for net benefit used in decision curve analysis. These papers are sparse on the rationale for doing so. We aim to assess the relation between sampling variability, inference, and decision-analytic concepts.

**Methods and results:**

We review the underlying theory of decision analysis. When we are forced into a decision, we should choose the option with the highest expected utility, irrespective of p values or uncertainty. This is in some distinction to traditional hypothesis testing, where a decision such as whether to reject a given hypothesis can be postponed. Application of inference for net benefit would generally be harmful. In particular, insisting that differences in net benefit be statistically significant would dramatically change the criteria by which we consider a prediction model to be of value. We argue instead that uncertainty related to sampling variation for net benefit should be thought of in terms of the value of further research. Decision analysis tells us which decision to make for now, but we may also want to know how much confidence we should have in that decision. If we are insufficiently confident that we are right, further research is warranted.

**Conclusion:**

Null hypothesis testing or simple consideration of confidence intervals are of questionable value for decision curve analysis, and methods such as value of information analysis or approaches to assess the probability of benefit should be considered instead.

## Highlights

• Several papers have recently proposed methods to calculate *p* values and 95% confidence intervals for net benefit. These have not clearly explained how such statistics should be used when evaluating a decision curve.

• Traditional decision theory dictates that we choose the option with the highest expected utility, irrespective of uncertainty.

• Sampling variability is relevant for understanding the value of further research.

• Value of information and the probability of benefit are how sampling variability can be appropriately incorporated in a decision-analytic context.

## Background

Decision curve analysis was introduced in 2006 [1] as a decision-analytic method for the evaluation of diagnostic tests and prediction models. It follows a classic approach to the evaluation of a classification method [2]. The key advance involves the concept of threshold probability (*p*_*t*_): the minimum predicted probability of an event at which a decision-maker, such as a doctor or patient, would opt for an intervention. This *p*_*t*_ is used both to determine whether a patient is classified as positive (if predicted probability *p̂* ≥ *p*_*t*_) versus negative (*p̂* < *p*_*t*_) and to weight the value of true positive vs. false positives, with the latter multiplied by the odds at *p*_*t*_ to calculate a net benefit. Decision curves display the net benefit of a diagnostic test or prediction model against the default options of intervening on all or no patients across a range of reasonable values for *p*_*t*_.

The advantage of decision curve analysis is that, in contrast with the traditional metrics of discrimination or calibration, it can inform the decision of whether to use a prediction model in clinical practice. For example, Nam et al. [3] compared two models for predicting the risk of prostate cancer on biopsy (“PCPT” vs. “Sunnybrook”) in men with elevated prostate-specific antigen (PSA). The Sunnybrook model had better discrimination (area-under-the curve [AUC] of 0.67 vs. 0.61) but was associated with some miscalibration. This leaves the clinician unsure of whether to use either model for helping decide whether to biopsy: does the miscalibration of the Sunnybrook model offset its higher discrimination? Is an AUC of 0.61 for the PCPT high enough? The decision curve gave a much clearer picture: neither model had a higher net benefit than the clinically reasonable default strategy of biopsy in all men with elevated PSA unless the threshold probability was very high (above 30%). There are obviously few men who would need a 30% risk of cancer before they opt for biopsy.

Decision curve analysis is now widely used in the empirical literature, with close to 2000 papers a year using the term in their abstract. It has also been recommended in editorials in several major journals including JAMA, BMJ, *Journal of Clinical Oncology* and the *Annals of Internal Medicine* [4–7]. Decision curve analysis is included in the TRIPOD statement for reporting of multivariable models [8]. Naturally, a secondary methodologic literature has developed, including reconceptualizing net benefit using the framework of decision regret [9] and relative utility [10], extension to time-to-event data [11] and integrating the net benefit function [12].

A number of papers have proposed methods for calculating confidence intervals for net benefit, as well as hypothesis tests to compare net benefit between two models or between a model and an alternative strategy such as assuming that all patients are positive. These include work by the original authors [11] and a number of recent papers proposing alternative formulations [12–15]. An interesting feature of this literature is that there is sparse discussion of how hypothesis testing or confidence intervals for decision curves are informative, under what circumstances they should be used or how they should be interpreted. There seems to be an implicit assumption that, because testing of hypotheses and estimating uncertainty of estimates is key in the field of statistics, and decision curve analysis is a statistical approach, we should report *p* values and 95% CI for net benefit. In the current paper, we aim to explore the relationship between sampling variability, inference, and decision-analytic concepts. We show that application of inference for net benefit is generally harmful and we propose a framework for the role of uncertainty in relation to decision curve analysis.

## Methods and results

### Proposals for confidence intervals and hypothesis tests for net benefit

We reviewed recent texts that propose methods for calculating confidence intervals or inference statistics on net benefit. Zhang et al. [12] presented a bootstrap method for inference on net benefit without any introduction or discussion; Sande et al. [15] similarly give no clear justification for inference on net benefit in the “Introduction” section of their paper. When discussing the motivating example, they claim that statistical testing of net benefit allows us “to compare models more rigorously”, but do not further explain what they mean by “rigor”. Pfeiffer and Gail introduce inference on decision curves as part of a well-developed discussion on different approaches to net benefit estimation. However, their exact justification for doing so appears to be that “we have not seen analytical statistical methods for putting confidence intervals [on net benefit] at a given threshold.” [14]. In contrast, Marsh et al. specify a clear role for estimating confidence intervals for net benefit, saying that these help determine whether a “biomarker warrants [further research], does not demonstrate clinical potential, or that more data are needed” [13]. That said, no clear methodology was specified for moving from a particular net benefit result to a particular conclusion, for instance, a given confidence bound including a certain value would indicate further research. In an editorial, Kerr et al. recommend estimates of uncertainty to researchers for a similar reason, and argue, quite reasonably, that policy makers “need some quantification of uncertainty”. But while they state that recommending confidence intervals for net benefit “does not prescribe how policy makers must use them in every instance”, the literature lacks any guidance on how confidence intervals should be interpreted in different scenarios [16].

We found no instances in the methodological literature where a specific decision curve is shown with and without confidence intervals and the authors make a clear and compelling argument how a decision, such as whether to use the model in practice, would be beneficially enhanced by this information. This includes the work by the original authors [11], who argue that confidence intervals might be of value “where a well-accepted clinical practice would be changed”, without providing an illustrative example. Pfeiffer and Gail provide as a motivating example prediction of breast cancer, but do not explain how the confidence intervals presented around the decision curve should be interpreted [14]. In their commentary, Kerr et al. say that “confidence intervals have heightened importance when current policy is treat-none” [16] but do not give a specific worked example. Given that many thousands of papers that have presented a decision curve analysis of empirical data without quantification of uncertainty, there would surely be empirical examples where presentation of confidence intervals would have improved interpretation of the findings, if this was indeed the case.

### Decision-analysis and inference

As a toy example, imagine that a statistician must catch a bus to get to the train station. There is a choice of getting a bus that goes downtown, or one that starts by heading crosstown, and there is not much to choose between them, such as one route having a better view, additional seating, or a more comfortable ride. For the sake of simplicity, we will also assume that there are no other options (such as a taxi) and that the train always leaves on time. Fortunately, some data are available on typical journey times for each route at the appropriate time of day and the statistician is able to calculate that they will get to the station on time 70% of the time on the downtown bus versus 67% of the time for the route that starts crosstown. The risk difference is 3%, with a 95% CI − 13% to 7%, *p* = 0.6. Under traditional decision theory, the statistician should take the downtown bus on the grounds that they would be more likely to catch their train.

This example illustrates two general features of decision-analytic thinking. First, *a decision has to be made*: the statistician has to take one bus or the other and, given the lack of statistical difference in times, if they do not take the “pick the winner” approach, they are left with flipping a coin. This contrasts with classical hypothesis testing, where there is nothing forcing us to draw a conclusion: we can avoid any statement about whether the crosstown or downtown bus is faster, stating only that the null hypothesis was not rejected and more data are required.

Second, *the size of the difference does not matter*: if the risk difference was only 0.5%, we should still take the downtown bus. In traditional statistical estimation problems, the size of an estimate is important because it has to be weighed against other estimates. The most obvious example would be a drug trial want to know how much better the drug is than placebo because we want to know if its benefits, say, the degree to which it reduces pain, offset its potential harms, side-effects, financial costs and risks. A decision analysis attempts to include all relevant considerations into a single estimate. The bus route example is somewhat limited, because we specified that there was no difference between the two routes other than time. In a more typical decision analysis, all possible benefits and harms of a particular decision are put on the same scale, typically a utility scale. The approach with the highest score is chosen, irrespective of the size of the difference. In the case of a decision analysis of a diagnostic test, for instance, we could consider not only the harms and benefits of true and false positive and negatives, but the cost, inconvenience and any harms of the test itself [7].

The toy example of the bus routes does not include a third critical feature of decision-analysis, which is that it *often involves multiple alternatives*. In the prostate biopsy example, we can biopsy all men with elevated PSA, none of the men, biopsy based on the Sunnybrook model or use the PCPT model instead. In contrast, hypothesis testing is binary—we reject or fail to reject a null hypothesis—and 95% CI are calculated either for a single estimate or the difference in two estimates.

Hypothesis testing has historically had a limited role in decision analysis [17]. Decision analyses rarely, if ever, report *p* values. They do commonly include sensitivity analyses where input estimates are varied, typically by considering different estimates reported in the literature or by using a “tipping point” approach, identifying how much an estimate would have to differ in order for the optimal decision to change. As a typical example, Packer et al. created a decision tree for the use of steroids with pregnant women at risk of preterm birth with concurrent respiratory infection [18]. Inputs to the decision tree included the risk of intensive care unit (ICU) admission and the risk of maternal death with and without steroids, taking estimates from the literature. In a sensitivity analysis, the risk of ICU admission was varied across a wide range and it was found that risk on steroids would have to be 32%, rather than the central estimate of 22%, in order for steroids to be the less beneficial strategy. However, this difference in estimates was discussed not in terms of sampling variability (e.g., whether the 95% C.I. for the cited study included 32%) but in terms of patient heterogeneity, such as whether older women might be at higher risk. Other studies use probabilistic sensitivity analyses, which does incorporate uncertainty in parameter estimates associated with sampling variability. However, the result is not a 95% C.I. around a decision-analytic estimate nor a *p* value [19].

### Potential harms hypothesis testing in decision analysis: interpreting prediction models

**Fig. 1:**
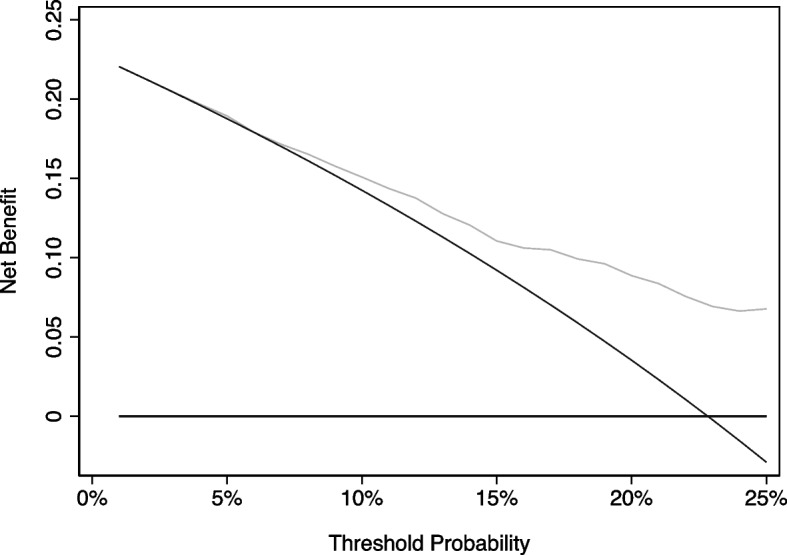
Decision curve for a hypothetical biopsy study. *N* = 600. Thick black line: treat none. Thin black line: treat all. Thin grey line: treat according to prediction model

To explore inference on the net benefit of a prediction model, we will use as an example the scenario described above where there is a prediction model for high-grade prostate cancer in a group of patients who are currently subject to biopsy. The prevalence in biopsied patients is 25%, meaning that large numbers of patients undergo unnecessary biopsy. We specify the range of reasonable threshold probabilities as 5% to 25%, with one widely accepted threshold being 10%. Let us assume that we conducted a large (*n* = 600) external validation and the model performs well according to current expert views, with an AUC of 0.76 and almost perfect calibration. The decision curve is shown in Fig. 1. The net benefit at the common threshold of 10% for the model is 0.1674 vs. 0.1574 for “biopsy all”. The net number of patients avoiding biopsy is calculated as the difference in net benefit × the reciprocal of the odds at the threshold probability = (0.1674–0.1574) × (1–0.1) ÷ 0.1 × 100 = 9 patients per 100 avoiding biopsy. A typical conclusion from such a curve is that the model is clinically useful: use of the model in the clinic would improve outcome by substantially reducing the number of patients exposed to the unpleasant biopsy procedure while not missing too many high-grade cancers among those with risks below 10%. A further discussion of how decision curve analysis should be used to inform clinical decision-making is given in the Appendix section.

**Fig. 2:**
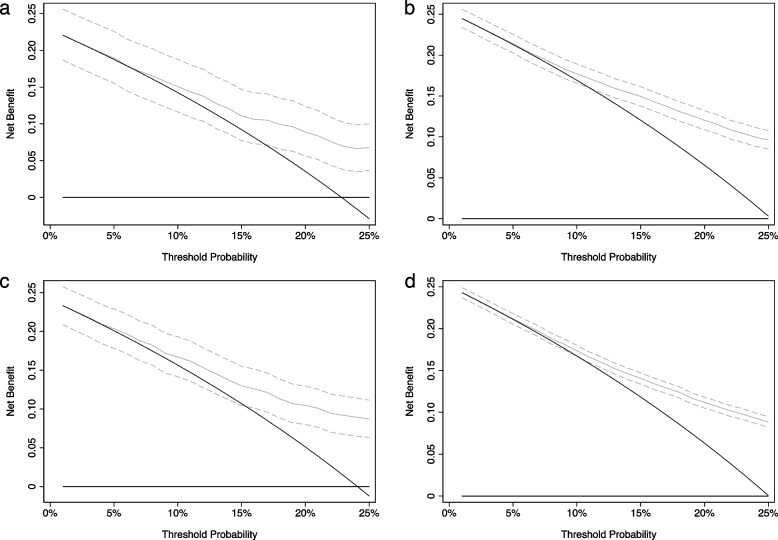
Decision curves for a hypothetical biopsy study, with 95% C.I. **a ***N* = 600 (same as Fig. 1). **b ***N* = 1200; **c ***N* = 5000; **d ***N* = 20,000. Thick black line: treat none. Thin black line: treat all. Thin grey line: treat according to prediction model. Dashed grey lines: 95% CI for treated according to prediction model

Figure 2a shows the decision curve with 95% CI which includes poorer net benefit than the “biopsy all strategy”. It is easy to see how this could be interpreted as demonstrating that the model could lead to harm and should therefore be seen as unproven. Figure 2b–d shows that this conclusion would not really change even if sample size was doubled (using the same parameters), or increased tenfold; it is only if sample size is 20,000 that the 95% C.I. do not cross the decision curve of “biopsy all” at the key threshold of 10%, and even then, benefit is not shown at the lower bound in the 5–9% range.

Comparing the lower bound of one decision curve with the central estimate of another, such as the decision curve for treat all, has been seen in the methodologic literature but is a questionable approach because of the correlation between curves. This is easily illustrated by thinking in terms of the bootstrap: if a bootstrapped sample by chance has a lower prevalence, this will lower the net benefit of both the model and of the treat all strategy.

A more valid approach would be to give 95% CI for both the difference in net benefit between a model and treat all, and for the difference in net benefit between the model and treat none, the latter being equivalent to the 95% C.I. for the net benefit of the model. In the case of the model shown in Fig. 1, the lower bound of the 95% CI for the difference in net benefit between treat all and treat depending on the model is − 0.0004 (*p* = 0.058) at a probability threshold of 10% and − 0.008 (*p* = 0.8) at 7.5%, but 0.009 (*p* = 0.0052) at 15%. It is likely that these findings would be considered to cast doubt on the value of the model. Only with a sample size four or five times larger than the original would we have reasonable power to reject the null at a threshold of 10%. For the threshold of 7.5%, the study would need to be about 20 times larger (*n* = 12,000). For a threshold of 5%, even a study with 250,000 patients would have low power to reject the null. Doubts would thus be raised about a prediction model that we would currently view as being of clear benefit to patient care. The power problem is even more extreme for the comparison between two models, where differences are smaller than between a single model and reference strategies. If, for instance, we assume that the difference between two models is half of that compared to a model and treat all, this would entail a sample size of 10–12,000 for the 10% threshold, 50,000 for the 7.5% threshold and many millions for the 5% threshold. This is therefore not simply a case of a method requiring more patients that researchers would otherwise prefer, it is making research completely infeasible.

Note that our critique of hypothesis testing is purely with respect to its application to decision-analytic metrics. We are not engaging in, and are indeed suspicious of, arguments that frequentist statistics are universally problematic [20].

### Appropriate use of hypothesis testing and uncertainty for net benefit and decision curve analysis

We have shown that requiring statistical significance for net benefit—either directly or implicitly by using confidence intervals—would dramatically raise the bar for supporting the use of prediction models. In many cases, it would make research infeasible by requiring sample sizes in the millions. Even where research would be possible, a strategy of only using models with statistically significant improvements in net benefit would delay implementation of effective strategies and hence lead to worse clinical outcomes in medicine as a whole compared to a strategy of using models where the central estimate of net benefit was superior.

Our view is that the role of considering uncertainty in decision curve analysis is to guide further research. In our bus example, the statistician would be right to take the downtown bus, but at the same time it would be wise to acknowledge that the decision might not be the right one. Specifically, on completing the trip, the statistician might well gather more data on bus times to use the next time there was a need to choose between the two bus routes.

In short, we not only want to know what decision to make, but how much confidence we should have in that decision. The problem is that *p* values are not informative on that point and confidence intervals are not directly linked to the ordinary meaning of the degree of “confidence” we have in a particular decision. It might be tempting to use a simplistic algorithm of seeing whether the *p* value for the difference in net benefit between a model and treat all or treat none is less than a conventional one-sided α of 0.025 and then making a binary conclusion of good or poor confidence in the decision, with the latter leading to calls for further research. However, we should be cautious about such a reflexive and incompletely argued approach. Firstly, there are finite financial and intellectual resources for medical research: calling for more research on one topic, say, evaluation of a model for cancer biopsy, means calling for *less* research on another, say, research on new biomarkers that could improve cancer prediction. Moreover, and critically, calls for further research are often used to avoid practical decisions. Most notoriously, the drumbeat of “further research” has been used to avoid curbs on tobacco and pollution, and there are clear parallels in clinical medicine: take, for instance, the position that cancer screening should be curtailed pending as yet, unplanned massive and extremely long-term studies powered for the endpoint of overall mortality [21]. One can easily imagine routine evaluations of prediction models becoming routine calls for further research that the *p* value for the difference in net benefit is ≥ 0.05 at one or other threshold probability.

On the other hand, there would appear to be cases where a call for further research would be justified. Take, for instance, where net benefit is highest for a cancer biopsy model across the relevant threshold probabilities, and consider the following three situations:

- The lower bound of the 95% CI around the difference in net benefit between the model and both treat all and treat none excludes 0 for the entire range of relevant thresholds;
- The lower bound includes a difference in net benefit less than zero, but only trivially so;
- The lower bound indicates a clear possibility of harm, that is, net benefit substantially lower than treat all or treat none for at least some thresholds.

In all three cases, we would follow traditional decision theory and recommend using the model in clinical practice: on the day after the paper is published a patient will present to a clinic with the risk factors for the cancer and a doctor will have to decide whether or not to biopsy—they cannot postpone that decision until another research paper is published—and the best decision is the one with the highest expected utility. However, in case (c), more external validation data is required to reduce uncertainty to an acceptable level. Note that we are leaving aside the issue of clinical impact studies, which are sometimes warranted to evaluate empirically the effect of a model on patient outcomes in practice.

Value of information analysis provides an attractive quantitative methodology for decisions about allocating research resources [22]. In a typical study, an analyst constructs a decision tree for a given medical decision (such as whether to use a certain drug), estimates how uncertainty in the inputs (such as the absolute risk reduction of the drug) affects cost-effectiveness (often in terms of the cost per quality adjusted life year) and then calculates the value of reducing uncertainty by a given amount for one or more of the inputs. The expected value of information is greater when the possible range of outcomes is high and the number of patients affected large: we should be more ready to fund a clinical trial if we are currently unsure whether a drug does a lot of good or a lot of harm in treating a common disease than if the possible outcomes of a drug for a rare disease varied between small and moderate benefit.

Recently, Sadatsafavi et al. have reported an approach for the net benefit of a model in a training data set based on the “Expected Value of Perfect Information” (EVPI). A Monte Carlo method is used to estimate the net benefit of a model if the true coefficients were known; the ratio of the observed net benefit of the current model to the hypothetical net benefit of an optimized model is informative of the value of further research [23]. Depending on whether this ratio is high or low, investigators should, respectively, move to external validation or conduct further studies on the model before external validation [23].

Further methodological work is ongoing to develop comparable approaches for the key scenario where decision curves are applied to an external validation data set [24]. In particular, there is a need to compare non-parametric (bootstrap-based), parametric and model-based approaches for calculating EVPI. Moreover, it remains to be determined how to interpret multiple EVPI estimates across the range of reasonable probability thresholds.

Value of information analysis is relatively complex and has not been widely applied in the context of prediction model research. An alternative is to calculate the probability that the model of interest is the superior strategy, compared to competing strategies (such as treat all, treat none, a diagnostic test or a competing model). A probability close to 1 means that there is little doubt that the model is of value; a probability just about 0.5 would indicate that although decisions taken with the model are likely to lead to better patient outcome than a strategy of treating all or no patients, there is considerable doubt associated with the model and further research is required. This approach bears similarities with cost-effectiveness acceptability curves [25] and stochastic league tables [26] in health economics, and the selection probability function for biomarkers [27].

Several other approaches have been reported in the literature. One is to conduct Bayesian meta-analysis of data from multiple studies or multiple centers, which may be from heterogenous settings where problems with model calibration may be expected [28]. In this case, it is interpreted as the probability that the model would be the best option, with the highest net benefit, in any randomly selected new setting. Even with very wide and overlapping credible and prediction intervals around the net benefit curves, this probability can be close to 1 at a wide range of thresholds.

## Conclusions

In decision analysis, we compare reasonable alternatives, and hypothesis testing will often be one of the criteria for deciding what counts as reasonable. An obvious example would be a decision analysis comparing two drugs, one which had higher efficacy, and the other lower toxicity. A conclusion that expected utility is greater for one of the two drugs will not generally include hypothesis testing, but the choice of drugs to enter into the decision analysis undoubtedly will. We would only consider drugs for the decision analysis that have been shown in clinical trials to be of benefit, and analyses of those trials undoubtedly involve inference statistics.

For the case of a decision curve analysis of a prediction model, we would argue that good statistical evidence favoring the model is paramount. While there are no widely agreed criteria as to what counts as “good statistical evidence”, many statisticians would be worried about a prediction model where most of the predictors were not significantly associated with outcome, or where the 95% CI for the C-index includes 0.5. It is not hard to create simulated data sets for such scenarios with apparently favorable decision curves, similar to prediction models based on noise variables [29].

Conversely, incautious use of hypothesis testing on net benefit in decision curve analysis would be harmful, changing the conventional interpretation of much prediction research and requiring infeasibly large sample sizes. There are no compelling examples where hypothesis testing would importantly change the conclusion of a decision curve analysis to a more intuitively correct finding. Sampling variability might indicate the need for further research. However, this should not change the conclusion of a decision curve based on the central estimate of net benefit, which guides us on whether a prediction model is likely to improve patient outcome. Inference or simple consideration of confidence intervals are of questionable value in this context, and more sophisticated methods, such as value of information analysis or approaches to assess the probability of benefit, should be considered.

## Data Availability

Not applicable.

## Appendix

### How should decision curve analysis be used to inform clinical decision-making?

A typical conclusion of a decision curve analysis is that basing clinical decisions on a prediction model will lead to better outcomes than any alternative strategy. This would seem to suggest that such a decision curve can be used directly to determine clinical practice, that is, once the paper has been published, clinicians should start making the relevant clinical decision using the prediction model in every relevant case. There are both intrinsic and extrinsic reasons why this may not be appropriate.

With respect to intrinsic influences, decision curve analysis does not always consider the complete range of influences on a given decision. For instance, in the biopsy example given in the main paper, the threshold probabilities are based on the clinician’s views about the relative harms and benefits of biopsy including pain, risk of infection and the risk of cancer progression. It may not consider financial costs of a biopsy, social costs of the biopsy (e.g. time off work for the patient) or resource constraints (e.g. availability of office space or MRI slots). Hence readers will often have to interpret the results of a decision curve in the light of factors that were not included in the analysis.

We characterize “extrinsic” influences on the interpretation of a decision curve analysis as similar to affecting interpretation of any clinical research paper. In brief, it is not the case that once a research paper is published, then practice universally changes. If, say, a randomized trial finds clinically and statistically significant differences between a new treatment and conventional care, the authors will conventionally conclude that patients should be offered the new treatment. Yet clinical implementation is rarely immediate and may not happen at all. Clinicians or policy makers may take into account the availability of other treatments, biological plausibility, replication studies and logistical concerns. A favorable decision curve analysis is subject to similar considerations. Net benefit for a model may be higher than for all alternatives across the full range of reasonable threshold probabilities, but clinicians or policy makers might rationally decline to implement if a similar model is already available, plausibility is questionable (e.g. predictors in the model are not widely validated), replication studies are missing or it would be difficult to incorporate the model into the clinical workflow smoothly.

Applicability to a local population is an additional key consideration before clinical implementation. This consideration holds similarly for implementation of results of a clinical trial of a new treatment or decision curve analysis of a prediction model. A clinician or policy maker may decline to recommend a treatment even if demonstrated as effective in a randomized trial if there are important differences between the population they are responsible for in comparison to the population evaluated in the trial; a similar position might be taken with respect to the decision curve analysis of a prediction model. An issue of particular pertinence for prediction modeling concerns minority populations. It is not at all uncommon for a prediction model to be validated in a cohort with limited minority representation and this may raise important questions about the properties of the model in such populations.
