# Correction: Decision curve analysis: confidence intervals and hypothesis testing for net benefit

**DOI:** 10.1186/s41512-025-00188-6

**Published:** 2025-03-30

**Authors:** Andrew J. Vickers, Ben Van Calster, Laure Wynants, Ewout W. Steyerberg

**Affiliations:** 1 Department of Epidemiology and Biostatistics, Memorial Sloan Kettering Cancer Center, New York, NY USA; 2 Department of Development and Regeneration, KU Leuven, Louvain, Belgium; 3 Department of Biomedical Data Sciences, Leiden University Medical Center, Leiden, the Netherlands; 4 EPICentre, KU Leuven, Louvain, Belgium; 5 Department of Epidemiology, CAPHRI Care and Public Health Research Institute, Maastricht University, Maastricht, Netherlands

**Correction: Diagn Progn Res 7, 11 (2023)**

**https://doi.org/10.1186/s41512-023-00148-y**

Following publication of the original article [1], the authors reported an error in the spelling of author Ben Van Calster

The incorrect author name is: Ben Van Claster

The correct author name is: Ben Van Calster
